# Flash glucose monitoring system in patients with type 1 diabetes in healthcare center in Brazil: real world data from a short-term prospective study

**DOI:** 10.20945/2359-3997000000540

**Published:** 2022-12-01

**Authors:** Alessandra S. M. Matheus, Juliana Brazil Fontes Pascoal, Carolina Alves Cabizuca, Lucianne Righeti Monteiro Tannus, Rafael Seno Guimarães, Diogo Menezes Ferrazani Mattos, Roberta Arnoldi Cobas

**Affiliations:** 1 Hospital Universitário do Estado do Rio de Janeiro Unidade de Diabetes Departamento de Clínica Médica Rio de Janeiro RJ Brasil Departamento de Clínica Médica, Unidade de Diabetes, Hospital Universitário do Estado do Rio de Janeiro, Rio de Janeiro, RJ, Brasil; 2 Universidade do Estado do Rio de Janeiro Ciências Médicas Rio de Janeiro RJ Brasil Ciências Médicas, Universidade do Estado do Rio de Janeiro, Rio de Janeiro, RJ, Brasil; 3 Universidade Federal Fluminense Departamento de Engenharia de Telecomunicações Programa de Pós-graduação em Engenharia Elétrica e de Telecomunicações Niterói RJ Brasil LabGen/MidiaCom, Programa de Pós-graduação em Engenharia Elétrica e de Telecomunicações, Departamento de Engenharia de Telecomunicações, Universidade Federal Fluminense, Niterói, RJ, Brasil

**Keywords:** Flash glucose monitoring system, FreeStyle Libre, glycemic control, time in range, glycated hemoglobin, continuous glucose monitoring, hypoglycemia

## Abstract

**Objectives:**

To evaluate the alternate use of flash glucose monitoring (FGM) with self-monitoring blood glucose (SMBG), in patients with type 1 diabetes (T1D).

**Materials and methods:**

Two weeks of open FGM (P2), both preceded (P1) and followed by 2 weeks (P3) of SMBG with a blinded FGM system. Mean absolute relative difference (MARD) was calculated by (|FGMi − SMBGi|) / SMBGi, where it was a paired data sample.

**Results:**

In total, 34 patients were evaluated. Time in range (TIR) did not change between P1 and P2. In 12 patients (35.3%), TIR increased from 40% at P1 to 52% at P2 (p = 0.002) and in 22 (64.7%), TIR decreased or did not change. FGM use resulted in decreased % time spent in hypoglycemia (<70 mg/dL) (6.5% vs. 5.0%; *p* = 0.005), increased % time spent in hyperglycemia (>180 mg/dL) (44.5% to 51%; *p* = 0.046) with no significant change in % TIR. The proportion of patients who reached sensor-estimated glycated hemoglobin (eA1c) < 7% decreased from 23.5% at P1 to 12.9% at P2, *p* = 0.028. For the whole sample, the MARD between the two methods was 15.5% (95% CI 14.5-16.6%). For normal glucose range, hyperglycemic levels and hypoglycemic levels MARD were 16.0% (95% CI 15.0-17.0%), 13.3% (95% CI 11.5-15.2%) and 23.4% [95% CI 20.5-26.3%)], respectively.

**Conclusion:**

FGM after usual SMBG decreased the % time spent in hypoglycemia concomitant with an undesired increase in % time spent in hyperglycemia. Lower accuracy of FGM regarding hypoglycemia levels could result in overcorrection of hypoglycemia.

## INTRODUCTION

Type 1 diabetes (T1D) is a chronic disease that requires individualized therapeutic intervention associated with self-management tools, such as regular and constant glucose monitoring. However, achieving ideal glycemic and metabolic control to minimize micro- and macrovascular complications remains a major challenge, especially for patients on complex insulin therapy regimens. According to the results from the BRAZDIAB study, a Brazilian multicenter study carried out in the public health system including 3,466 patients with T1D, only 13.2% of adults and 23.2% of children met the goal for glycated hemoglobin (A1c) (1,2).

According to large studies such as the Diabetes Control and Complications Trial (DCCT), A1c levels are considered the best parameter for assessing glycemic control and the incidence of diabetes-related chronic micro- and macrovascular complications (3). However, A1c levels do not identify daily fluctuations in blood glucose, including glycemic variability or hypoglycemia, making intensive daily glucose monitoring important for optimizing treatment (4,5).

New methods for monitoring glucose include detecting glucose in the interstitial fluid through a continuous glucose monitoring (CGM) or flash glucose monitoring (FGM) system, in parallel with traditional ambulatory self-monitoring of blood glucose (SMBG). The FGM system has emerged recently as a useful, practical, and well-proven tool in randomized clinical trials for intermittent and real-time blood glucose monitoring (6,7). Therefore, the available technology promotes self-care, more assertive decisions in adjusting insulin doses by the attending medical team, and consequently reductions in glycemic variability and its negative consequences on the metabolic control of patients with T1D (8,9).

The FGM system has been proved in the Al Hayek's prospective study to improve clinical parameters once the patients had been switched from the fingerprick method to FGM over 12 weeks in children and teenagers with T1D (10). Moreover, a recent study demonstrated a significant and sustained reduction in HbA1c over 6 months in a pediatric population with FGM (11). However, none of those studies have alternated the use of FGM with SMBG.

The primary aim of this study was to evaluate the clinical impact of the alternate use of FGM with SMBG in patients with T1D in a public healthcare unit in Rio de Janeiro, Brazil. Also, we aimed to identify the characteristics of patients who benefited the most from using FGM in terms of improvements to TIR.

## MATERIALS AND METHODS

This prospective cohort study was conducted at the Diabetes Outpatient Clinic of the State University of Rio de Janeiro between May 2019 and September 2020. The inclusion criteria were T1D patients older than 4 years, regular follow-up at the outpatient clinic, and stable insulin regimen for at least 3 months. T1D was clinically defined according to the American Diabetes Association criteria (12). The exclusion criteria were individuals currently using a CGM system, allergy to any medical adhesive device, renal replacement therapy (hemodialysis or peritoneal dialysis), recent or actual use of glucocorticoids for any pathology, pregnancy, or women planning to become pregnant in the forthcoming months.

The study was approved by local center's ethics committee and CAEE “*Certificado de Apresentação para Apreciação Ética*” certified at Plataforma Brasil was 07626319.0.0000.5259. Written informed consent for the study was obtained from all of the patients aged 18 years or older, and from the parents or guardians of the patients younger than 18 years old.

The following variables were assessed by a questionnaire or medical records: gender, current age, age at diabetes diagnosis, duration of diabetes, self-reported ethnicity, education level, smoking status, exercise frequency, height (cm), weight (kg), insulin dose (IU/kg), type of insulin (human or analog), SMBG frequency, blood pressure, and heart rate. Body mass index (BMI) was determined by dividing weight (kg) by the square of height (m^2^). Economic status was defined by the Brazilian Economic Classification Criteria (13), and the following economic classes were considered for this analysis: high, medium, low, and very low.

A1c was evaluated at the baseline and at the end of the 2-month protocol period. Samples of 1.5 μL of blood were obtained by puncturing the digital pulp (*point of care*) using the Boronate method with the Alere Afinion HbA1c AS100 Analyzer equipment kit, which provides results after 3 minutes (VR = 4.0 to 15%). This method has been validated and meets all National Glycohemoglobin Standardization Program (NGSP) A1c performance criteria. (14)

### FGM protocol

The FreeStyle™ Libre Pro system (Abbott Diabetes Care, Alameda, California) is a glucose reader that allows intermittent glucose monitoring in the interstitial fluid through a sensor applied under the skin, which lasts 14 days. Each patient's glucose monitoring was assessed for a continuous period of 2 months (4 sensors). The participants attended the Diabetes Clinic every 14 days to change the sensor and upload data to a computer. The collected data were sent retroactively to the health professional who assisted the patient, and the information on glycemic variability, time in range (TIR), percentage of time in hypoglycemia and hyperglycemia, and frequency of hypoglycemia were analyzed. First, the patients had a 14-day run-in period for the FGM, for adaptation and instruction. This was important because no patient had used it before. Those who did not adapt to the run-in period could not continue in the study. Afterward, the first period started (P1), during which we masked the sensor by placing adhesive tape over the reader display and instructed the patients to scan 8 times/day at the same time when they used SMBG, even without seeing the results, to capture all the baseline parameters provided by the FGM during the usual care on SMBG. During this blind period, patients were instructed to adjust their insulin therapy according to SMBG, performed 8 times/day (before and after meals).

**Figure 1:**
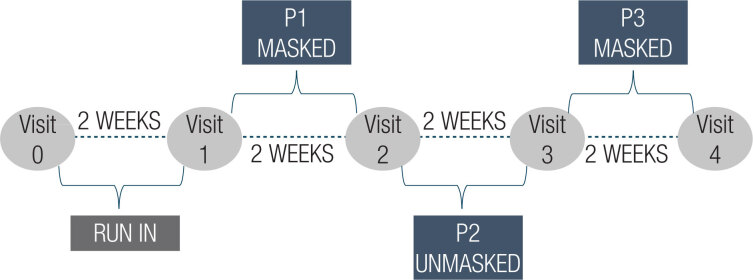
Study design.

The second period (P2) was designed to evaluate if FGM, as compared to the intermittent results of SMBG, could affect the patients’ behavior and attitudes as well as their management of insulin doses. Afterward, in the third period (P3), we repeated the masked use of the sensor to evaluate if the impact of previous FGM use was sustained after interruption and return to usual SMBG, possibly allowing for intermittent use of the FGM (Figure 1).

Run in: first exclusive use of flash glucose monitoring system period; P1: first blind period; P2: second exclusive use of flash glucose monitoring system period; P3: last blind period.

The FGM parameters analyzed were percentage of FGM use (defined as the percentage of time FGM was active and recommended > 70% of data from a period of 14 days), sensor-estimated A1c (eA1c; automatically calculated by FGM), TIR (70-180 mg/dL), and percentage of time in different glycemic ranges: level 2 hypoglycemia (<55 mg/dL), level 1 hypoglycemia (<70 mg/dL), level 1 hyperglycemia (>180 mg/dL) and level 2 hyperglycemia (>250 mg/dL) (5). These parameters were evaluated at the end of P1 (SMBG effect), P2 (FGM effect), and P3 (intermittent use effect).

The FGM parameters analyzed were percentage of FGM use (defined as the percentage of time FGM was active and recommended > 70% of data from a period of 14 days), sensor-estimated A1c (eA1c; automatically calculated by FGM), TIR (70-180 mg/dL), and percentage of time in different glycemic ranges: level 2 hypoglycemia (<55 mg/dL), level 1 hypoglycemia (<70 mg/dL), level 1 hyperglycemia (>180 mg/dL) and level 2 hyperglycemia (>250 mg/dL) (5). These parameters were evaluated at the end of P1 (SMBG effect), P2 (FGM effect), and P3 (intermittent use effect).

At each clinical visit, the medical staff adjusted the insulin doses based on SMBG measurements at the end of P1 and P3 and according to the ambulatory glycemic profile provided by FGM at the end of P2.

### Statistical analysis

The data are shown as mean ± standard deviation (SD) or as median [interquartile range (IQR)] for continuous variables and as numbers (relative frequencies) for discrete variables. The numeric variables were compared using parametric and non-parametric tests, either paired or not, as appropriate. The chi-square and Fisher tests were used to compare the frequencies of the categorical variables. The statistical analyses were performed with Statistical Package for Social Sciences (SPSS) for Windows (version 25.0; IBM, Armonk, NY). A two-sided *p-value* less than 0.05 was considered significant.

The accuracy of FGM was evaluated using the mean absolute relative difference (MARD), a variable calculated as the difference between the interstitial glucose values obtained from FGM and blood glucose, as measured with the usual SMBG, at the same time. MARD is given by (|FGMi − SMBGi|) / SMBGi, where it is a paired data sample. The MARD results are presented with 95% CIs, calculated considering a Student's t-distribution. Pearson's correlation coefficient was used to evaluate the relationship between FGM and SMGB. An ordinary least squares regression was deployed to calculate the function for associating the FGM measurement with the expected SMBG measurement. The regression coefficients help to show where a dependent relationship exists between FGM and SMBG (15). For graphical purposes, Clarke error grid analysis was applied to measure the consensus between FGM and SMBG. The statistical analyses of the concordance and the correlations between FGM and SMBG were performed with the Scipy 1.3.3, Sklearn 0.22.2, and Pandas 0.25.3 libraries running on top of the Python 3.8.10 programming language.

## RESULTS

We enrolled 39 participants between May 2019 and August 2020. Of these participants, five (12.8%) were excluded before completing the study (1 withdrew informed consent, 1 was not followed-up during the second period, 2 presented diabetic ketoacidosis caused by infection during the second period, and 1 lost 2 sensors due to inadequate sensor care at the first period). Five sensors stopped working before 14 days (mean of 10 days use). Two children felt pain upon the sensor's insertion. Six participants presented bleeding at the site of the sensor's insertion. We replaced 19 total sensors over the time of the study.

The baseline characteristics of the studied population are shown in Table 1. A1c levels decreased by 0.2 ± 0.4%, from 8.4 ± 1.3% at baseline to 8.2 ± 0.9% at the end of the study (*p* = 0.05).

**Table 1:** Baseline characteristics of the studied population

Characteristic		N = 34
Male, n (%)		20 (58.8)
Age (years)		16 (8-30)
Age at diagnosis (years)		12 (6-17)
Diabetes duration (months)		54 (14-192)
Scholarship (years), n (%)		
	1 y	5 (14.7)
	2 y	9 (26.5)
	3 y	0
	4 y	4 (11.9)
	5 y	9 (26.5)
	6 y	3 (8.8)
	7 y or above	4 (11.8)
Economic status (* s), n (%)		
	High	0 (0)
	Medium	9 (26.5)
	Low	24 (70.6)
	Very low	1 (2.9)
Age group, n (%)		
	Children (4 to 12 years)	12 (35.3)
	Teenagers (13 to 18 years)	6 (17.6)
	Adults (>18 years)	16 (47.1)
BMI (kg/m^2^)		20.9 (16.6-25.5)
Ethnicity (Caucasian), n (%)		19 (55.9)
SMBG/day		3.8 ± 1.2
Insulin treatment:		
	Basal insulin (UI/kg/day)	0.35 ± 0.2
	Bolus insulin (UI/kg/day)	0.34 ± 0.19
	Total daily Insulin (UI/kg/day)	0.69 ± 0.31
	Type of basal insulin (NPH/Long-acting analogs), n (%)	15 (44.1)/17 (50)
	Type of bolus insulin (Regular/Rapid-acting analogs), n (%)	13 (38.2)/21 (61.8)
	CSII, n (%)	2 (5.9)
Mean A1c, %		8.4 ± 1.3

Data are shown in mean ± SD, median (IQR range) or n (%).

* ABEP's classification:

*Associação Brasileira de Empresas de Pesquisa*; BMI: body mass index; SMBG: self-monitoring blood glucose; CSII: continuous system of insulin infusion; A1c: glycated hemoglobin.

The mean FGM use for each 14-day period was 90.7%, 92.1%, 93.0% and 92.5%, respectively.

### Analysis comparing P1 (SMBG effect/FGM blinded) and P2 (FGM effect)

The mean number of sensor scans per day was 7.1 ± 2.1 for P1 and 9.6 ± 3.4 for P2.

#### Time in range

**Figure 2:**
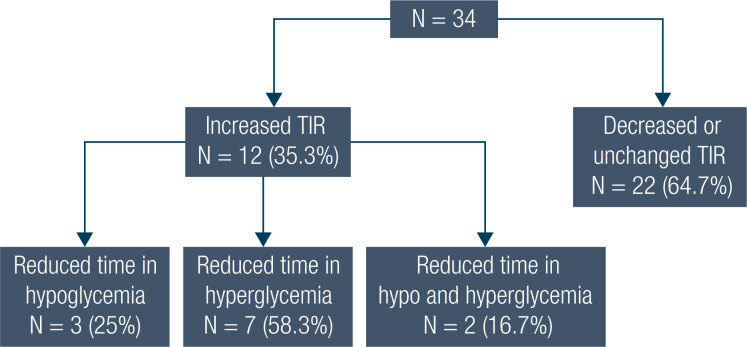
Changes in time in range (TIR) between P1 and P2

Overall, TIR did not change between P1 and P2 (Table 2). In 12 patients (35.3%), TIR increased from 40% at P1 to 52% at P2 (*p* = 0.002) and in 22 (64.7%), TIR decreased or did not change (Figure 2). The factors associated with this improvement were as follows: male gender (10 [83.3%] *vs.* 9 [42.9%], *p* = 0.024), lower total daily insulin at baseline (0.42 [0.29-0.69] *vs.* 0.77 [0.59-0.98] IU/kg/day, *p* = 0.018), and lower A1c at baseline (8.2 [7.3-8.3]% *vs.* 8.8 [8.3-9.5]%; *p* = 0.037), when compared to those patients that did not improve, respectively. No difference was found in ethnicity, physical exercise practice, current age, diabetes duration, age at diagnosis, baseline SMBG frequency, type of basal or bolus insulin, difference between insulin dose (IU/kg) from P2 to P1, economic status and number of scans in P2.

**Table 2:** Glycemic control and treatment data from P1 (SMBG effect) and P2 (FGM effect)

		P1 (SMBG effect)	P2 (FGM effect)	p value
eA1c %		7.73 ± 1.0	7.98 ± 0.99	0.134
	Goal eA1c < 7% n (%)	8 (23.5)	4 (12.9)	**0.028**
TIR (70-180mg/dL)				
	Time spent (%)	43 [37.3-55.8]	43 [33-55]	0.484
	Goal TIR > 70% n (%)	3 (8.8)	1 (3.1)	0.063
Hypoglycemia level 1 (<70 mg/dL)				
	Time spent (%)	6.5 [4.0-13.5]	5.0 [2.0-10.0]	**0.005**
	Goal hypoglycemia level 1 < 4% n (%)	6 (17.6)	15 (46.9)	0.006
Hypoglycemia level 2 (<55 mg/dL)				
	Time spent (%)	3.0 [1.0-7.5]	2.0 [1.0-4.0]	**0.031**
	Goal hypoglycemia level 2 < 1% n (%)	4 (12.1)	7 (21.9)	0.212
Hyperglycemia level 1 (>180 mg/dL)				
	Time spent (%)	44.5 [35.0-54.3]	51 [41.0-64.0]	**0.046**
	Goal hyperglycemia level 1 < 25% n (%)	5 (14.7)	2 (6.5)	**0.013**
Hyperglycemia level 2 (>250 mg/dL)				
	Time spent (%)	18 [6.5-24.5]	21 [11.0-31.0]	0.121
	Goal hyperglycemia level 2 < 5% n (%)	5 (15.2)	1 (3.7)	0.185
FGM use (%)		93.5 [88.5-96.3]	95 [93-97]	0.063
Insulin IU/kg/day				
	Basal	0.42 [0.2-0.5]	0.45 [0.2-0.6]	**0.020**
	Bolus	0.32 [0.2-0.4]	0.35 [0.2-0.4]	0.238
	Total	0.77 [0.4-1.1]	0.77 [0.4-0.9]	0.341

Data are shown in mean ± SD, median (IQR range) or n (%). SMBG: self-monitoring blood glucose; FGM: flash glucose monitoring; P1: period 1; P2: period 2; eA1c: sensor-estimated glycated hemoglobin.

The proportion of patients who reached eA1c < 7% decreased from 23.5% at P1 (SMBG effect) to 12.9% at P2 (FGM effect), *p* = 0.028 (Table 2).

#### Hypoglycemia

The percentage of time spent in level 1 hypoglycemia (<70 mg/dL) decreased from 6.5 [1.0-4.0]% at P1 to 5.0 [2.0-10.0]% at P2 (*p* = 0.005). (Table 2). The proportion of participants who reached the goal for percentage of time in hypoglycemia level 1 of < 4% increased from 17.6% at P1 to 46.9% at P2 (*p* = 0.006) (Table 2).

Percentage of time spent in hypoglycemia showed no association with insulin dose (total, basal, or bolus). Patients who used regular human insulin presented higher percentages of time with hypoglycemia < 70 mg/dL in P1, but not in P2, than those on rapid-acting insulin analogs did (13 [6-19.5] % *vs.* 6 [3.5-11]%, *p* = 0.035). No difference was observed during P1 or P2 in the percentage of time in hypoglycemia < 70 mg/dL, regarding type of basal insulin used (intermediate human insulin [NPH] or long-acting insulin analogs).

#### Hyperglycemia

The percentage of time spent in hyperglycemia level 1 increased from 44.5 [35.0-54.3] % at P1 to 51 [41.0-64.0] % at P2 (*p* = 0.046), and the number of patients who reached less than 25% of time spent on level 1 hyperglycemia decreased from 5 (14.7%) at P1 to 2 (6.5%) at P2 (*p* = 0.013) (Table 2). In 10 patients (32.3%), the percentage of time spent in hyperglycemia level 1 decreased.

#### Insulin dose

Basal daily insulin dose increased from P1 to P2 (0.42 [0.20-0.55] to 0.45 [0.22-0.56] IU/kg/day, *p* = 0.02). No significant change occurred in total insulin dose (*p* = 0.08) or in daily insulin bolus (*p* = 0.27) from P1 to P2 (Table 2).

#### Analysis comparing P2 (FGM effect) and P3 (intermittent use effect): impact of a possible intermittent use of FGM

After the transition from P2 to P3, the percentage of time spent in hypoglycemia level 1 increased (8 [4-13]% *vs.* 5 [2-10]%, *p* = 0.019). Fewer patients reached the goal of percentage of time spent in hypoglycemia level 1 < 4% (7 [21.2%] *vs.* 15 [46.9%], *p* = 0.032) and more patients reached the eA1c target of < 7% [11 (33.3%) *vs.* 4 (12.9%), *p* = 0.012]. No association existed between percentage of time spent in hypoglycemia in P2 or P3 and insulin dose (total, basal, or bolus) or type. No statistically significant difference was observed in the other glycemic control parameters.

#### Accuracy of FGM when compared to SMBG

Twenty-six of the 34 patients were included in the sensor performance evaluation because this subgroup had 14 days of complete SMBG data (1705 total measurements). For all of the measurements over 14 days, FGM had an adequate correlation with SMBG (r = 0.90, *p* < 0.001) (Table 3). For the whole sample, the MARD between the two methods was 15.5% (95% CI 14.5-16.6%), and the highest discrepancy occurred in hypoglycemia level (23.4% [95% CI 20.5-26.3%)]. The MARD values for TIR and for hyperglycemic levels were 16.0% (95% CI 15.0-17.0%) and 13.3% (95% CI 11.5-15.2%), respectively. For hypoglycemia levels, the correlation with FGM was very low (0.159, *p* = 0.026). Regression coefficients for the entire sample, TIR and hyperglycemia levels were 0.87, 0.82, and 0.75, respectively, with a small 97.5% CI span (Table 3), showing a tight dependency between FGM and SMBG. However, for hypoglycemia level, the regression coefficient was 0.65 with a 97.5% CI spanning from 0.08 to 1.23. The large CI span reveals a discrepancy trend between FGM and SMBG while in hypoglycemia level.

**Table 3:** Comparison among FGM and SMBG

Clarke error grid analysis	Accurate %	Acceptable (benign) %	Erroneous %	Pearson correlation + linear regression FGM x SMBG
Overall (n = 26 patients/1,705 measurements)	96.60	0.76	2.63	0.90 (<0.001) 0.87 {0.85-0.89}
Glucose range < 70 mg/dL (N = 25 patients/146 measurements)	95.38	0.51	4.10	0.16 (=0.02) 0.65 {0.08-1.23}
Glucose range 70-180 mg/dL (N = 26 patients/813 measurements)	97.57	0.11	2.30	0.68 (<0.001) 0.82 {0.76-0.88}
Glucose range > 180 mg/dL (N = 26 patients/746 measurements)	98.12	1.73	0.15	0.74 (<0.001) 0.75 {0.70-0.80}

FGM: flash glucose monitoring (FreeStyle Libre); SMBG: self-monitoring blood glucose. [97.5% CI], (p value).

**Figure 3:**
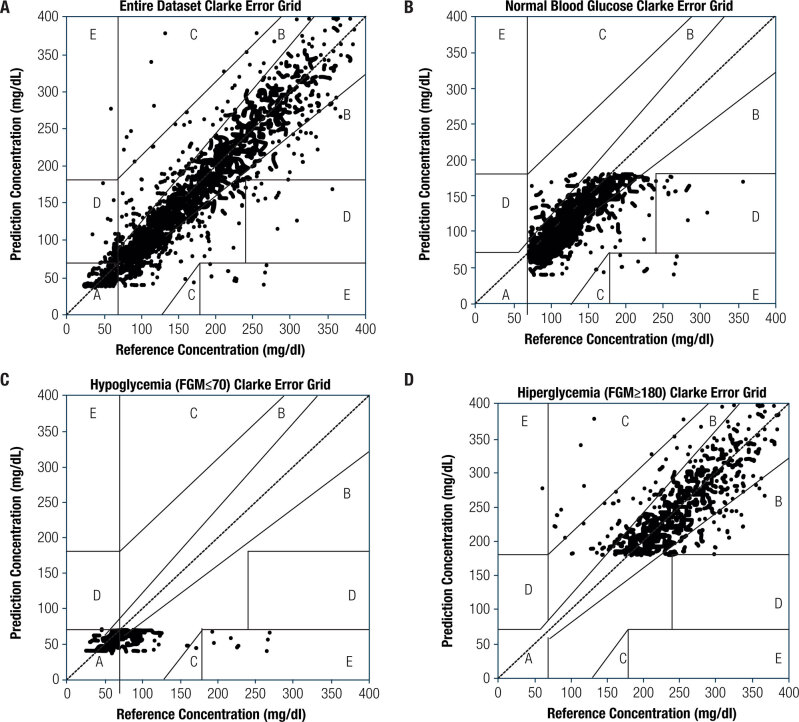
Clarke error grid analysis between flash glucose monitoring (FGM) and self-monitoring blood glucose (SMBG).

The Clarke error grid (Figure 3) showed that 96.6% of the sample pairs were in acceptable zones (78.3% in zone A and 18.3% in zone B) (Table 3) (Figure 3A). Regarding the glucose measurements for hypoglycemia level by FGM, 69.2% of the glucose pairs were in zone A, and 26.1% were in zone B, but 4.1% were in zone E (Figure 3B). Hence, 4.1% of the hypoglycemia pairs detected by FGM were within the normal glucose range according to the reference method (SMBG), at the same time. For TIR, Clarke Error Grid showed that 71.9% were in zone A, and 25.7% were in zone B, implying that just 0.1% of the glucose pairs were in error zone C (Figure 3C) and 2.3% were in zones D and E. For the hyperglycemia levels, 98.1% of the glucose pairs were in the acceptable zones (zones A and B), while 1.7% of the samples estimated by FGM were higher than the reference value (zone C upper), and 0.15% were estimated to be lower than the reference value (zone E lower) (Figure 3D).

Clarke error analysis between FGM within the entire database (**Figure A**), normal glucose range (**Figure B**), hypoglycemia (**Figure C**) and hyperglycemia **(Figure D**), according to the reference method (SMBG), at the same time.

## DISCUSSION

In the present study, short-term use of FGM after usual SMBG revealed that TIR decreased or did not change in the majority of patients and revealed a decreased percentage of time spent in hypoglycemia concomitant with an increased time spent in hyperglycemia but not TIR, as desired. We also showed lower concordance of the FGM with the hypoglycemia level compared to SMBG. To our knowledge, this is the first study to evaluate the clinical impact of FGM among adults and pediatric patients with T1D in a Brazilian public tertiary diabetes center.

The use of a blinded FGM made it possible to discover that patients used to spend long periods in previously undetected hypoglycemia. As expected, FGM use lowered the percentage of time spent in hypoglycemia, in agreement with the findings from the IMPACT study, in which 358 patients with T1D were randomized to either the FGM group or the control group (SMBG) for 6 months, also resulting in a decreased percentage of time spent in hypoglycemia at the end of study in the FGM arm (8). Thus, when unmasked, the continuous glucose monitoring by FGM possibly allowed for changes in patient behavior toward preventing and/or correcting the detected hypoglycemic episodes. However, the reduced percentage of time spent in hypoglycemia with FGM use occurred in parallel with an undesired increase in the percentage of time spent in hyperglycemia. These findings contrast with those of other studies that showed a lower percentage of time spent in hypoglycemia along with improvement in TIR without an increased percentage of time in hyperglycemia (16,17).

This study was not intended to directly assess FGM's performance when compared to SMBG because the accuracy of FGM has already been described elsewhere (7,18). Rather, we decided to explore concordance with SMBG, particularly for hypoglycemia levels. We found that patients who were considered to be within hypoglycemia level by FGM were, in fact, already in TIR as measured by SMBG. This can be explained by the higher percentage of discrepancy found in the Clarke error analysis in FGM hypoglycemia levels, the low correlation with SMBG in this glucose range, and the higher MARD found (23.4%). Thus, when therapeutic adjustments were made by study staff to reduce hypoglycemia, they shifted to the hyperglycemic range. Our results are in agreement with the study by Ji and cols., which showed 6% incorrect values in Clarke's error analysis (zones D + E) (7). Furthermore, the MARD we found for the entire sample was 15%, while Ji and cols. (7) found 10%.

Our results are similar to those found by Battelino and cols., who demonstrated that FGM use reduced time spent in hypoglycemia, in parallel to an increase in time spent in hyperglycemia (>180 mg/dL). However, differently from us, they observed increased TIR (19). These data led us to infer that patients may have overcorrected their hypoglycemic episodes, thus reinforcing the need to vigorously provide and reinforce diabetes education. The occurrence of hypoglycemia frightens most patients, even if asymptomatic. Moreover, the observation of downward trend arrows may trigger patient overcorrection (for example, by overfeeding) due to fear of hypoglycemia, leading to a deliberate maintenance of hyperglycemia. These data are in accordance with the findings from the FUTURE study, which reported decreased percentage of time spent in hypoglycemia and decreased TIR, along with an increased percentage of time spent in hyperglycemia level 1 after 1 year of follow-up of 1913 T1D patients using FGM (20).

TIR remained almost the same throughout the study. However, we identified that in 36% of patients, mostly males with lower baseline A1c levels and using lower insulin doses, TIR increased during FGM use, as compared with SMBG. These results are supported by the findings of a long-term study including 347 children and adolescents that showed a better glycemic outcome in only 35% of patients, after 1 year using FGM (21). In addition, female gender was independently associated with poor glycemic control in the BrazDiab study (2). Lower doses of basal insulin in males were associated with lower A1c levels, as shown in a retrospective study including 89 patients (22).

We observed a 0.2% decrease in A1c levels from baseline to the end of our study. This was similar to the study conducted by Campbell and cols. (16), which found a 0.3% reduction in A1c in 31 participants (children and adolescents) after multiple daily injections of insulin, at the end of eight weeks of follow-up. In addition, the findings from a recent meta-analysis of 25 real-world observational studies and randomized controlled trials including adults and children showed a 0.3% and a 0.33% A1c reduction for adults and children, respectively, within the first 2 months of continuous FGM use (4).

Adverse sensor-wear events were mild and tolerated, but they impacted the number of sensors used throughout the study. Early sensor detachment was the largest barrier of our study, especially in children. This finding was also the most encountered inconvenience in a study with 67 children (23). Moreover, these findings are consistent with the reported prevalence rates, especially in children in a short-duration study (9). A recent real-world study in a summer camp with 78 children showed that the mean duration of the sensors was 10.5 days and that 10 extra sensors were replaced in 8 children (24).

The strength of this study was that it included real-world data from a Brazilian tertiary public center. We chose to include patients with T1D, regardless of their glycemic control, gender, age, or insulin treatment regimen.

Finally, we must address some limitations. These include the short duration of the study, the relatively few participants in each age group (children, teenagers, and adults), and the lack of a control group with which to compare our results.

In conclusion, FGM use after usual SMBG revealed that in the majority of patients TIR decreased or did not change and revealed a decreased percentage of time spent in hypoglycemia concomitant with an increased time spent in hyperglycemia. Male gender, lower total insulin dose, and lower A1c at baseline were the factors associated with improved TIR in a subgroup of patients. The lower accuracy of FGM at lower glucose levels could have clinical effects in overcorrection of hypoglycemia. Further studies over a longer period are needed to assess whether patients would manage hypoglycemia events better with the help of FGM.

## References

[1] Gomes MB, Coral M, Cobas RA, Dib SA, Canani LH, Nery M (2012). Prevalence of adults with type 1 diabetes who meet the goals of care in daily clinical practice: a nationwide multicenter study in Brazil. Diabetes Res Clin Pract.

[2] Gomes MB, de Mattos Matheus AS, Calliari LE, Luescher JL, Manna TD, Savoldelli RD (2013). Economic status and clinical care in young type 1 diabetes patients: a nationwide multicenter study in Brazil. Acta Diabetol.

[3] The DCCT Research Group (1991). Epidemiology of severe hypoglycemia in the diabetes control and complications trial. Am J Med.

[4] Evans M, Welsh Z, Ells S, Seibold A (2020). The Impact of Flash Glucose Monitoring on Glycaemic Control as Measured by HbA1c: A Meta-analysis of Clinical Trials and Real-World Observational Studies. Diabetes Ther.

[5] Gabbay MAL, Rodacki M, Calliari LE, Vianna AGD, Krakauer M, Pinto MS (2020). Time in range: a new parameter to evaluate blood glucose control in patients with diabetes. Diabetol Metab Syndr.

[6] Calliari LEP, Krakauer M, Vianna AGD, Ram Y, Barbieri DE, Xu Y (2020). Real-world flash glucose monitoring in Brazil: can sensors make a difference in diabetes management in developing countries?. Diabetol Metab Syndr.

[7] Ji L, Guo X, Guo L, Ren Q, Yu N, Zhang J (2017). A Multicenter Evaluation of the Performance and Usability of a Novel Glucose Monitoring System in Chinese Adults With Diabetes. J Diabetes Sci Technol.

[8] Per Oskarsson, Antuna R, Geelhoed-Duijvestijn P, Krӧger J, Weitgasser R, Bolinder J (2018). Impact of flash glucose monitoring on hypoglycaemia in adults with type 1 diabetes managed with multiple daily injection therapy: a pre-specified subgroup analysis of the IMPACT randomised controlled trial. Diabetologia.

[9] Edge J, Acerini C, Campbell F, Hamilton-Shield J, Moudiotis C, Rahman S (2017). An alternative sensor-based method for glucose monitoring in children and young people with diabetes. Arch Dis Child.

[10] Al Hayek AA, Robert AA, Al Dawish MA (2020). Effectiveness of the Freestyle Libre Flash Glucose Monitoring System on Diabetes Distress Among Individuals with Type 1 Diabetes: A Prospective Study. Diabetes Ther.

[11] Leiva-Gea I, Martos-Lirio MF, Gómez-Perea A, Ariza-Jiménez AB, Tapia-Ceballos L, Jiménez-Hinojosa JM (2022). Metabolic Control of the FreeStyle Libre System in the Pediatric Population with Type 1 Diabetes Dependent on Sensor Adherence. J Clin Med.

[12] American Diabetes Association (2021). 16. Diabetes Advocacy: Standards of Medical Care in Diabetes-2021. Diabetes Care. Diabetes Care.

[13] Brazilian Association of Research Companies (2012). Brazilian Economic Classification Criteria.

[14] Torregrosa ME, Molina J, Argente CR, Ena J (2015). Evaluación de tres sistemas de determinación rápida de hemoglobina A1c para monitorización del control glucémico en pacientes con diabetes mellitus. Endocrinol Nutr.

[15] McGraw KO, Wong SP (1996). Forming inferences about some intraclass correlation coefficients. Psychol Methods.

[16] Campbell FM, Murphy NP, Stewart C, Biester T, Kordonouri O (2018). Outcomes of using flash glucose monitoring technology by children and young people with type 1 diabetes in a single arm study. Pediatr Diabetes.

[17] Dover AR, Stimson RH, Zammitt NN, Gibb FW (2017). Flash glucose monitoring improves outcomes in a type 1 diabetes clinic. J Diabetes Sci Technol.

[18] Leelarathna L (2018). Invited Review Flash forward: a review of flash glucose monitoring. Diabet Med.

[19] Battelino T, Nimri R, Dovc K, Phillip M, Bratina N (2017). Prevention of hypoglycemia with predictive low glucose insulin suspension in children with type 1 Diabetes: a randomized controlled trial. Diabetes Care.

[20] Charleer S, De Block C, Van Huffel L, Broos B, Fieuws S, Nobels F (2020). Quality of Life and Glucose Control After 1 Year of Nationwide Reimbursement of Intermittently Scanned Continuous Glucose Monitoring in Adults Living With Type 1 Diabetes (FUTURE): A Prospective Observational Real-World Cohort Study. Diabetes Care.

[21] Vergier J, Samper M, Dalla-Vale F, Ventura V, Baucher F, Joubert F (2019). Evaluation of flash glucose monitoring after long-term use: A pediatric survey. Prim Care Diabetes.

[22] Strich D, Balagour L, Shenker J, Gillis D (2017). Lower Basal Insulin Dose is Associated with Better Control in Type 1 Diabetes. J Pediatr.

[23] Massa GG, Gys I, Op ‘t Eyndt A, Bevilacqua E, Wijnands A, Declercq P (2018). Evaluation of the FreeStyle® Libre Flash Glucose Monitoring System in Children and Adolescents with Type 1 Diabetes. Horm Res Paediatr.

[24] Szadkowska A, Gawrecki A, Michalak A, Zozulińska-Ziółkiewicz D, Fendler W, Młynarski W (2018). Flash Glucose Measurements in Children with Type 1 Diabetes in Real-Life Settings: To Trust or Not to Trust?. Diabetes Technol Ther.

